# Perception of Antiretroviral Generic Medicines: One-Day Survey of HIV-Infected Patients and Their Physicians in France

**DOI:** 10.1371/journal.pone.0117214

**Published:** 2015-02-06

**Authors:** Christine Jacomet, Clotilde Allavena, Fleur Peyrol, Bruno Pereira, Laurence Morand Joubert, Haleh Bagheri, Laurent Cotte, Rodolphe Garaffo, Laurent Gerbaud, Pierre Dellamonica

**Affiliations:** 1 Service des Maladies infectieuses et tropicales, Hôpital Gabriel Montpied, CHU Clermont Ferrand, Clermont-Ferrand, France; 2 Service des Maladies infectieuses et tropicales, CHU Hôtel-Dieu, Nantes, France; 3 Santé Publique, EA 4681 PEPRADE, CHU Clermont-Ferrand, Clermont-Ferrand, France; 4 Service de Biostatistiques, CHU Clermont-Ferrand, Clermont-Ferrand, France; 5 Laboratoire de Virologie, INSERM-UMR_S 1136, CHU Saint Antoine, Paris, France; 6 Pharmacologie Médicale et Clinique, INSERM U1027, CHU Toulouse, Toulouse, France; 7 Service des Maladies infectieuses et tropicales, INSERM U1052, Hospices Civils de Lyon, Lyon, France; 8 Pharmacologie Clinique, CHU Pasteur, Nice, France; 9 Service des Maladies infectieuses et tropicales, CHU de l'Archet, Nice, France; David Geffen School of Medicine at UCLA, UNITED STATES

## Abstract

**Background:**

In the interest of cost effectiveness, switching antiretroviral brand name medications to generics is recommended in France since 2013. The study objective was to evaluate the perception of generics per se and antiretroviral generics in HIV-infected patients and their hospital physicians

**Methods and Findings:**

556 out of 703 (79%) adult HIV+ outpatients and 116 physicians in 33 clinics were included in a multicentric cross-sectional survey performed in September 2013. Patients completed a self-questionnaire on their perception and acceptability of generics. Physicians completed a questionnaire on their acceptability of switching antiretroviral to generic. Socio-demographic data, medical history and HIV history were collected. Among the 556 patients with a median HIV duration of 13 years, 77% were France native, 59% in active employment, 100% covered by social insurance, 95% on antiretroviral therapy. Seventy-six percent of the patients accepted generics and 55% trusted them overall. Antiretroviral generics were accepted by 44% of them but only by 17% if the pill burden was going to increase. The factor significantly associated with acceptability of antiretroviral generics was acceptance of generics per se (p<0.001). Among the 116 physicians following a median of 100 HIV-patients/year, 75% would prescribe generics, dropping to 26% if the combo had to be broken. Factors significantly associated with willingness to prescribe antiretroviral generics were the absence of concern regarding the chemical entity (OR = 0.33), being aware that the patient would accept generics for other pathologies (OR = 2.04) and would accept antiretroviral generics (OR = 1.94). No factor related to sociodemographic conditions, HIV status or comorbidities was associated with the acceptability of antiretroviral generics.

**Conclusions:**

Acceptability of antiretroviral generics in this French population was mostly dictated by the patient’s and physician’s knowledge and use of generics overall. It should be improved with an efficient information of both patients and physicians.

## Introduction

In France, recurrent health expenditure amounted to 243 billion Euros in 2012, i.e. 12% of the gross domestic product (GDP) [1]. Treatment and health product consumption represented three-quarters of this sum, which has increased by +2.2% per year for the past two years. This expenditure on health should continue to rise all the more so as the population ages. However, in the Social Security Finance Bill (PLFSS) for 2014, the rate of progression of the National Objective for Health Insurance Spending (ONDAM) was set at 2.4%, whereas 2.8% was set for 2013 [2]. Obliged like other governments to find a way to regulate pharmaceutical costs due to economic pressure on the healthcare system, the French State is taking action via its drug pricing policy, the rate of reimbursement or the financing of new drugs. It is also possible to encourage the prescription of generics or switch to them (2006 agreement concerning third-party payment for prescriptions when a generic presentation is accepted).

According to article 10.2 of European Directive 2001/83/CE, a generic drug is defined as a speciality presenting the same composition in terms of quality and quantity of active principles as the brand name medication. Qualification of a generic drug is based essentially on demonstration that the generic presents a pharmokinetic profile that is superimposable relatively to the brand name medication. The identical nature of pharmacokinetic properties is assessed according to the bioequivalence parameter, for which a confidence interval of between 20% and 25% is tolerated according to the European Medicines Agency (EMA) [3–4]. As early as 2010, WHO quoted under-utilisation of generic drugs as one of the reasons for less than optimum economical efficiency of healthcare systems. After studying the situation in seventeen middle-income countries, it was estimated that substitution could reduce costs by 60% on average [5]. In December 2012, the French Drug Safety Agency (ANSM) stated that "The policy concerning drugs is a major factor in the evolution of the healthcare system. In a context of the necessary rationalization of healthcare expenditure, in order to promote equal access to the best treatments and innovations in treatments, generic drugs appear to provide an answer" [6]. The manufacturer's selling price for generics is indeed around 50% lower than that of the brand name drugs [Calculation of the price of generic drugs including tax in 2013]. This "economically constrained environment" is where generic drugs belong and they are expected to develop in the years to come in France. Currently they enable over 2.4 billion Euros to be saved over the ongoing year.

It has been shown that patients suffering from chronic conditions may be ill at ease with substitution [7]. This could be the case for HIV-infected patients in developed countries. The first antiretroviral (ARV) drugs became available in 1987, and in 2013 the patents of several ARV drugs expired, as in the case of lamivudine, zidovudine, nevirapine and efavirenz. Furthermore, generic presentations of a number of ARVs used today will be available in a few years.

We therefore thought it was important to carry out a survey of the perception of ARV generics among HIV-infected individuals and their hospital specialists, in order to better understand the reasons for acceptability or reluctance against these drugs.

The main objective was to assess the perception of ARV generics by HIV-infected patients and their hospital specialists in France in 2013. The secondary objectives were to assess what patients and their physicians knew about generics, the degree of acceptation and confidence patients had concerning generics overall and specifically ARV generics, the perception of ARV generics by physicians and their prescription attitudes, along with the degree to which replies by patients matched those of their physicians.

## Methods

We carried out a multicentric cross sectional survey on "a given day" covering all HIV-infected patients attending on the busiest day in the week of 16 to 21 September 2013 in outpatient hospital departments.

The patient inclusion criteria were an age over 18 and being aware of HIV infection for over 6 months. The exclusion criteria were being unable to fill out the questionnaire, not being able to speak French and refusal to take part.

Data have been collected on an anonymous basis using four questionnaires. The first questionnaire covered demographic, social and medical data collected from the medical file. The second one was a "patient" self-questionnaire concerning awareness and perception of generics including ARV generics. The third one was a questionnaire for physicians concerning prescription of ARV generics for the included patient. The fourth one was a "physician" self-questionnaire concerning perception of generics overall.

All analyses were carried out using Stata software (version 13, StataCorp, College Station, USA). The population was described by number of subjects and associated percentages for categorical variables, and by means (and associated standard deviation) or medians (and interquartile range [IQR]) with respect to the statistical distribution for quantitative variables (Shapiro-Wilk test of normality). Comparisons between independent groups (patients accepting or not accepting generics, prescription or not of ARV generics by the physician) were made using the Student t-test or the Mann-Whitney test if the conditions for the t-test were not respected (with the Snedecor-Fisher test for homoscedasticity). Comparisons of qualitative parameters between independent groups were made by the Chi2 test or where appropriate by the Fisher exact test. In the event of multivariate conditions, logistic regression models were constructed in order to take account of variables considered as significant in univariate conditions (p<0.1) or clinically pertinent in view of the literature. The relative risk allowed was set at 5% for all tests.

The protocol was registered and received approval from the French Advisory Committee on Data Processing Related with Health Research (CCTIRS) and National Commission for Data Protection and Liberties (CNIL).

## Results

The survey was carried out in 33 hospital departments spread over France, and recruited 116 physicians who received 703 HIV-infected patients for a visit during the study period. 556 patients (79%) were included, of whom 160 patients (29%) came from 10 centers located in the IIe-de-France departments. Twenty-four (68%) of the centers were University teaching hospital centres.

The reasons for non-inclusion were being unable to speak French (N = 25), unable to fill out the questionnaire (N = 50), non-availability (N = 24), not interested (N = 32), or other (N = 15). There was no significant difference between patients included in the survey and those excluded with respect to sex, -75% versus 69% of men respectively (p-value = 0,58) or to age,—mean of 48.35 (±11.34) and 48.96 (±35,51) years old respectively (p = 0,89).

Table 1 shows the demographic and characteristics for the patients who took part in the survey. Most of them were men, France native, half of whom had their high school diploma or a higher one, and 57% were in employment. Almost all were covered by social security insurance and only 14% did not have top-up insurance. The median duration of HIV infection was 13 years and 95% of these patients were receiving ARV treatment, for a median duration of 11 years, with a HIV viral load lower than < 50 copies/ml for 79% of them. Sixteen percent were co-infected by VHC and/or VHB and 23% presented another comorbidity.

Physicians who took part in the survey had a median age 47 (IQR 37–54), were in practice for 18 years (IQR 10–26), following a median of 100 HIV-patients/year (IQR 50–200). Most of them (65%) worked in an Infectious Diseases department.

**Table 1:** Patients demographic and medical characteristics (N = 556).

		Percentage or median (IQR)
Country of birth	France	77%
	Africa	18%
	Europe	4%
	Other	2%
Highest level of education	No qualification	6%
	Primary school	9%
	Secondary school diploma	11%
	Initial professional diploma	24%
	High school diploma	16%
	University degree	34%
In employment	Full or part-time	57%
Social security coverage	General system	90%
	Universal health insurance coverage (CMU)	10%
	State medical assistance (AME)	0%
Additional coverage	Top-up insurance policy	78%
	CMU top-up coverage	8%
	None	14%
HIV duration		13 (5–21)
Risk factor for HIV transmission	Homo/bisexual	46%
	Heterosexual	38%
	Injecting drug use	10%
	Other	5%
CDC stage	A	55%
	B	20%
	C	24%
ARV treatment	yes	95%
	duration (years)	11 (3–16)
	> 6 months	85%
	< 6 months	10%
CD4	Most recent CD4 count/mm^3^	580 (420–799)
	Nadir/mm^3^	224 (103–240)
HIV viral load	< 50 copies/ml	79%
	>50 copies/ml < 6 months	2%
	>50 copies/ml > 6 months	19%
Comorbidity	None	60%
	Co-infection HCV	13%
	Co-infection HBV	6%
	Other chronic pathology	23%
	- Arterial hypertension	16%
	- Cardiovascular pathology	14%
	- Diabetes	5%
	- Kidney failure	4%
	- Cancer	2%
	- Other	5%
Hospital monitoring	Every month	10%
	Every 3 months	56%
	Every 6 months	33%
	Every year	1%
ARV delivery	Pharmacy in town	49%
	Hospital pharmacy	30%
	Both	21%

Concerning the knowledge the patients and physicians participating in the survey had, 54% of patients and 50% of physicians were aware of the 40 to 50% savings made thanks to generic ARVs and 50% and 53% of them respectively had a rough idea of the annual cost of ARVs reimbursement in France (approximately 800 million Euros/year). Most of the patients (62%) agreed with the definition of a generic drug and 83% had already received one. Finally, about half of all physicians ignored that an antiretroviral drug ranked at the 8^th^ place among medicines fully reimbursed by the National Health Insurance.

### Patients' questionnaire

Overall 409 patients (76%) accepted generics: 81% of them willingly and 19% of them because the National Health Insurance will directly charge the pharmacy for generics.

Among the 129 (24%) patients who did not accept them, the reasons given most frequently were lack of confidence (20%), refusal as a rule (16%), the fear they would be less efficient (16%) or fear of side effects (7%). In univariate analysis, factors associated with acceptability of generics *per se* by the patients were male gender, France native, presumed non-heterosexual route of transmission of HIV (p = 0.001), education level above high school diploma, non AIDS, agreeing with the definition of a generic drug and already having received a generic drug (Table 2). Multivariate analysis showed that the fact of agreeing with the definition of a generic drug (p<0.001) and having already received a generic drug (p = 0.02) remained statistically associated with acceptability of generics by the patients. Overall, if 138 (26%) patients expressed no opinion, 309 (55%) of them trusted generics—increasing to 76% and 58% if generics were recommended by the physician and the pharmacist, respectively.

**Table 2:** Factors associated with acceptance of generics *per se* by patients.

		Refusal	Acceptance	p-value
		N = 129	N = 409	
		N (%)	N (%)	
Male sex		80 (62.0)	321 (78.5)	<0.001
Age, mean (sd)		48.9 (10.9)	48.3 (11.5)	0.64
France native		46 (36.8)	79 (19.8)	<0.001
Living in the Ile-de-France region		90 (69.8)	295 (72.1)	0.60
Educational high school diploma or higher		17 (20.2)	67 (33.3)	0.04
In employment		63 (50.8)	235 (59.2)	0.10
Social security coverage		121 (94.5)	393 (96.3)	0.44
HIV duration ≥13ans		64 (51.2)	205 (51.8)	0.91
Heterosexual transmission		62 (53.9)	133 (34.9)	<0.001
CDC stage	A	64 (50.8)	222 (57.5)	0.01
	B	19 (15.1)	83 (21.5)	
	C	43 (34.1)	81 (21.0)	
Treatment duration		10.9 (7.0)	10.2 (7.1)	0.35
Current CD4 count ≥500/mm^3^		73 (58.4)	269 (67.1)	0.08
CD4 nadir ≥200/mm^3^		116 (92.8)	383 (95.5)	0.23
HCV status		18 (14.1)	49 (12.0)	0.64
HBV status		8 (6.3)	20 (4.9)	0.84
Cardiovascular comorbidities		13 (10.2)	59 (14.5)	0.35
All comorbidities		55 (42.6)	146 (35.7)	0.16
Patient agreeing with the definition of a generic		28 (21.7)	310 (76.2)	<0.001
Patient having already received generics		66 (55.5)	368 (91.1)	<0.001
Patient foregoing third party payment		16 (33.3)	24 (14.7)	0.01
Patient accepting generics *per se*		8 (7.9)	230 (79.6)	<0.001

Switching ARV brand name medications to generics would be accepted by 44% of the patients—but only by 17% if the pill burden was going to increase—and was denied by 29% of them for reasons of confidence (65%), as a rule (36%), by fear of effectiveness (38%), of adverse side effects (36%), or the fact they are disturbing (17%). No opinion was expressed by 27% of the patients. In univariate analysis, factors associated with acceptance of ARV generics by the patients were male gender, France native, presumed non-heterosexual route of HIV transmission, AIDS free, agreeing with the definition of a generic drug, having already received a generic drug, foregoing third party payment, and accepting generics *per se* (Table 3). Multivariate analysis showed that the only criterion associated with acceptance of ARV generics was acceptance of generics *per se (*p<0.001).

**Table 3:** Factors associated with acceptance of ARV generics by patients.

		Refusal	Acceptance	p-value
		N = 158	N = 241	
		N (%)	N (%)	
Male sex		99 (62.7)	198 (82.2)	<0,001
Age, mean (sd)		48.9 (10.8)	49.2 (12.1)	0.79
France native		108 (69.7)	192 (82.4)	0,003
Living in the Ile-de-France region		47 (29.8)	57 (23.6)	0,18
Educational high school diploma or higher		26 (23.4)	34 (24.8)	0,80
In employment		75 (48.7)	136 (58.1)	0,07
Social security coverage		152 (96.8)	233 (96.7)	0,15
HIV duration ≥13ans		78 (50.6)	129 (55.4)	0,36
Heterosexual transmission		70 (48.6)	71 (32.1)	0,005
CDC stage	A	73 (48.0)	122 (54.5)	0,03
	B	28 (18.5)	54 (24.1)	
	C	51 (33.5	48 (21.4)	
Treatment duration (years)		11.3±6.8	10.6±7.1	0,29
Current CD4 count ≥500/mm^3^		101 (66.5)	151 (64.0)	0,62
CD4 nadir ≥200/mm^3^		143 (94.1)	222 (94.1)	0,99
HCV status		18 (11.5)	29 (12.0)	0,19
HBV status		8 (5.1)	12 (5.0)	0,86
Cardiovascular comorbidities		22 (14.0)	33 (13.7)	0,75
All comorbidities		62 (39.2)	91 (37.8)	0,77
Patient agreeing with the definition of a generic		31 (19.8)	224 (93.3)	<0,001
Patient having already received generics		104 (70.8)	217 (91.9)	<0,001
Patient foregoing third party payment		20 (30.3)	11 (13.6)	0,03
Patient accepting generics *per se*		59 (38.8)	230 (96.6)	<0,001

### Physicians’questionnaire

Among the 116 participating physicians, the strongest arguments in favor of ARV generics were protection of the healthcare system (67%) and access to treatments for everyone (59%), ahead of the fact that they could finance innovation (27%). The weakest arguments were concerns regarding efficacy (47%), the occurrence of adverse side effects (53%), and less active and less measured chemical entity (26% and 21% respectively). Furthermore, physicians would agree to prescribe ARV generics if they were aware of bioequivalence data (65%) and/or clinical study data (42%); 19% would not prescribe ARV generics despite ANSM (Agence Nationale de Sécurité du Médicament) guideline. ARV generics would be proposed by physicians when initiating a treatment (47%) after the first line (42%), with the same number of doses (53%) or the same number of pills (53%). One percent of the physicians would never prescribe any ARV generics.

Regarding the prescription of ARV generics for a patient (545 responses), 75% of physicians would be favorable but only 26% of them if the combo had to be "broken". Prescription of ARV generics would not modify the schedule of follow-up for 61% of situations. The main reasons for non ARV generics prescription were the fears relative to modification of formulation (54%), being aware that the patient would not accept (51%) (i.e. those patients who declined generics overall, lack of understanding, risk of non-compliance, anxiety, depressive symptoms), tolerance (39%) and efficacy (39%). In univariate analysis, factors associated with prescription of ARV generics by the physician were a younger age, a smaller number of patients in the physician active file, the absence of concern regarding the chemical entity, galenical formulation or adverse side effects, and understanding the contribution of generics for the preservation of the healthcare system (Table 4). Physicians were also more prone to prescribe ARV generics for male patients, currently in employment, agreeing with the definition of a generic, accepting generics for other pathologies and accepting ARV generics. Multivariate analysis showed that factors associated with willingness to prescribe generic ARVs were the absence of concern regarding the chemical entity (OR = 0.33, IC_95%_ = [0.17–0.63]), being aware that the patients would accept generics for other pathologies (OR = 2.04, IC_95%_ = [1.06–3.93]) and would accept ARV generics (OR = 1.94, IC_95%_ = [1.03–3.66])).

**Table 4:** Factors associated with prescription of ARV generics by physicians.

		Refusal	Acceptance	p-value
		N = 138	N = 407	
		N (%)	N (%)	
Physicians' profiles (N = 116)				
Age, mean (sd)		49.8 (10.3)	47.5 (10.6)	0,03
Experience, mean (sd)		21.1 (10.9)	19.1 (10.8)	0,05
Smaller number of patients in his active file, mean (sd)		125.6 (93.9)	85.1 (84.7)	<0,001
In practice in the Ile-de-France region		112 (27.5)	295 (72.5)	0,49
Fear of less measured chemical entity		66 (50.0)	205 (50.4)	0,94
No fear of less active chemical entity		74 (56.1)	134 (32.9)	<0,001
No fears related with galenical formulation		70 (53.0)	149 (36.6)	0,001
No fear of undesirable side effects		70 (53.0)	166 (40.8)	0,01
Role in preservation of healthcare system		77 (58.3)	273 (67.1)	0,07
Low price statement		106 (80.3)	320 (78.6)	0,68
Agreement concerning financing of innovation by the savings made		20 (15.2)	88 (21.6)	0,11
Awareness of costs for health insurance system		50 (62.5)	157 (51.8)	0.09
Awareness of savings achieved by a generic		33 (41.8)	134 (45.1)	0.60
Patients' profiles (N = 545)				
Male sex		91 (65.9)	312 (76.7)	0,01
Age, mean (sd)		49.8 (11.1)	48.1 (11.5)	0,12
Country of birth = France		104 (77.0)	298 (71.1)	0,64
Highest level of education > Baccalaureate		21 (22.3)	63 (24.8)	0,54
In employment		69 (50.0)	232 (59.3)	0,004
Social security coverage		130 (94.2)	392 (96.6)	0,27
HIV dating back ≥13ans		76 (56.7)	192 (48.6)	0,11
Means of contamination	Homosexual/Bisexual	58 (45.7)	187 (49.7)	0,44
	Heterosexual	52 (40.9)	153 (40.7)	
	Injecting drug use	17 (13.4)	36 (9.6)	
CDC stage	A	62 (47.7)	223 (57.3)	0,16
	B	31 (23.8)	76 (19.5)	
	C	37 (28.5)	90 (23.1)	
Duration of treatment ≥11ans		83 (60.1)	220 (54.1)	0,21
CD4 count ≥500/mm^3^		88 (64.2)	256 (64.3)	0,98
CD4 nadir ≥200/mm^3^		130 (94.9)	377 (94.7)	0,94
Co-infection HCV		20 (14.5)	46 (11.3)	0,09
Co-infection HBV		10 (7.3)	19 (4.7)	0,19
Cardiovascular comorbidity		26 (18.8)	48 (11.8)	0,11
All comorbidities (without any)		27 (19.6)	62 (15.2)	0,06
Patient agreeing with the definition of a generic		66 (48.5)	269 (66.9)	<0,001
Patient having already received generics		105 (80.1)	327 (83.6)	0,36
Patient accepting generics for another pathology		81 (60.5)	319 (81.2)	<0,001
Patient accepting ARV generics		38 (27.5)	200 (50.6)	<0,001

When physician and patient were independently in agreement in refusing ARV generics (Table 5), factors associated with this refusal were fears related with tolerance (previous ARV brand name side effects), related with patient acceptance (previous refusal of ARV generics, history of depression or anxiety) and patient refusal generics for another pathology. Multivariate analysis showed that previous ARV brand name side effects (OR = 0.20, IC_95%_ = [0.05–0.88]), previous refusal of generics (OR = 0.21, IC_95%_ = [0.05–0.87]) and refusal generics for other pathologies (OR = 15.0, IC_95%_ = [2.80–80.16]) were independently associated with refusal of ARV generics by both the patient and his physician.

**Table 5:** Reasons for physician's refusal (N = 138) associated with patient's refusal to use ARV generics.

Reasons quoted by the physicians	Patient Refusal	Patient Acceptance	p-value
	N = 56	N = 82	
	N (%)	N (%)	
Fears concerning effectiveness			
- Not suitable due to viral load	5 (9.3)	6 (16.7)	0,22
- Not suitable due to CD4 count	1 (1.8)	0 (0.0)	0,20
- Does not fit the treatment strategy	22 (40.7)	18 (48.7)	0,37
- Length of time under treatment	15 (27.8)	14 (38.9)	0,24
- Risk of resistance occurring	13 (24.1)	5 (13.9)	0,13
Fears related with tolerance			
- previous ARV brand name side effects	18 (42.9)	4 (12.1)	0,004
Fears related with changes in formulation	16 (40.0)	22 (61.1)	0,066
Fears related with patient acceptance			
- Often unwilling to accept treatment decisions	16 (36.4)	6 (17.7)	0,07
- Already expressed refusal	31 (63.3)	4 (12.1)	<0,001
- Difficulties in understanding	10 (18.2)	7 (19.4)	0,54
- Compliance less good	18 (33.3)	13 (36.1)	0,84
- Anxiety	27 (58.7)	10 (32.3)	0,02
- Depression	18 (40.9)	4 (13.3	0,02
Patient who does not accept generics for another pathology	15 (37.5)	33 (94.3)	<0,001

## Discussion

This study that has evaluated the perception of generics in an HIV-infected population and in their specialist physicians shows that the acceptability of ARV generics is mainly driven by the knowledge and the use of generics overall. Although both patients’ and HIV specialist physicians’ allegations against generics appeared to be highly inadequate, only one third of patients and one fourth of physicians are openly opposed to generics. Switching ARV brand name medications to ARV generics was mostly accepted if the patient accepted generics overall and if the pill burden was unchanged.

This study is the first one to describe the perception of generics on the part of HIV infected patients in France. The participation of sites all over the country is a striking point. Certainly, this survey has been only performed in hospitals and not with general practitioners, but in France in 2013 HIV infection is most often managed in hospitals care unit. Only repeated prescriptions can be given by the general practitioner and are limited to less than one year. Job sites were based on voluntary participation of centres that could have biased the selection of physicians as well as the recruitment of patients. Furthermore subjects who did not speak French or that were unable to read the questionnaire were excluded of the survey. Nevertheless, the enrolled population appeared to be representative of the HIV-infected population in France, since the socio-demographic and medical characteristics, such as the percentage of patients under treatment and the immuno-virological criteria, were similar to those in the recent VESPA 2 survey [8].

The responses to a few simple economic questions revealed that some patients and physicians are not well-informed with little difference between them, and this lack of basic knowledge is true for half of them. This may be linked to the fact that 100% coverage by the healthcare system eclipses the concept of the costs involved for society in general.

Only two out of three patients agreed with the definition of "generic drug". But two out of three questioned physicians declared that they would prescribe generics for their patients when bioequivalence studies become available, despite the fact that all generics need to comply with this criterion to be marketed. There may be a gap in physicians' knowledge and understanding about generic medicines due to the fact they are highly sceptical [9]. This scepticism is close to that of the 203 general practitioners who were surveyed in March 2012, when 37% of them did not agree with the following postulate: "generic medicines are medicines that have been proven to be effective and safe" [10]. This attitude is often found in earlier publications, indicating the degree of suspicion of a non-neglible portion of healthcare specialists concerning the process for marketing generics [11–12]. It is true that the French National Academy of Pharmacy issued an opinion and recommendations in 2012 along with a reminder of a certain number of points, in particular that the guarantee of quality and safety of generic pharmaceuticals is supported by the marketing authorization file lodged by manufacturers, and the checks made on it by the healthcare authorities (ANSM) [13]. But the recent report by the French National Academy of Medicine entitled "The place of generics for prescription" says the following: "Bioequivalence between the reference product and a generic does not mean there is automatically treatment equivalence, in particular when one generic preparation is substituted for another". (…) " The fact that production sometimes takes place in distant places with a multitude of sub-contractors makes on-the-spot checks difficult. Consequently, pharmaceutical checks on the finished product are all the more important, especially given the fast-developing market of counterfeit preparations, notably in China"[14]. These factors could probably create and maintain doubts about these medicines, strengthened by opinion leaders [15].

Concerning the patients, 55% trusted generics. When the physician had a positive attitude, this figure rose to 76%. In the study of patients aged over 50 by R. Ringuier et al, 30% of patients had already refused a generic preparation at least once, and significantly more if they had not been given information by their general practitioner [16].

One out of three patients did not accept ARV generics spontaneously, and those who did accept them (provided the pill burden remains the same) were those who accepted generics *per se*. Actually, a certain number of worries are expressed concerning generic drugs and there are debates about their quality, whether they are as efficient as the brand name medication, and concerning implementation of pharmaceutical substitution. Any medicine is at the same time remedy and poison. Drugs suffer from this ambivalent social representation and are symbolic of benefits but also dangers (adverse effects, dependency) [17]. Indeed drugs are delivered to care and cure patients. On the other hand patients may overcome a negative potency including somatic and psychological side effects that at times can reach to death. Patients may not understand what the relationship is between a generic and a brand name medication and do not know what requirements generics should fulfill, resulting in suspicions: "Because the collective representations see them as the result of healthcare cost control rather than of medical research, the negative aspect of the dual nature of medication tends to predominate in the perception of generic drugs meaning they are seen as chemical products and consequently liable to be toxic. This interpretation explains why people tend to be afraid of taking them" [18].Moreover, this acceptance appeared to be lower in the female population of foreign origin, notably African, for whom generic medicines are often synonymous with counterfeits, which is indeed sometimes true [19]. No criterion related with other socio-demographic or medical conditions (immunodepression, co-infection by hepatitis viruses, other comorbidities, etc) seemed to hinder acceptance of ARV generics by the patients. Criteria such as age [20], pathology, [21], therapeutic class [22], and also complexity of treatment and the risk of confusion related with changes in references [23], and level of education [24] played no role in our study whereas they were examined in other studies. The financial impact of substitution [25], notably depending on insurance coverage [26], was identified as primordial in other healthcare systems, but this was not researched in France due to fact that HIV infection treatment is fully reimbursed by the French social security system.

All in all 75% of physicians were in favour of prescribing generic ARVs mainly for patients who already accept generics for another pathology and accept ARV generics. This demonstrates that the patients' past experience has a strong impact on the prescription of new therapeutic classes, as already underlined by Allenet [27].

Physicians agreed with the patients not to prescribe generics for those who had had side effects with the brand name medication: the introduction of a generic product in treatment management could upset the patients' attitudes to their treatments, resulting in feelings that they are less effective or have side effects which the patients possibly experienced with the brand name medication but hadn't recognised as such [6, 28].

These results show that no criteria related with sociodemographic conditions or medical conditions (immunodepression, co-infection by viral hepatitis, other comorbidity, etc) had constrictive influence on the prescription of ARV generics. The bond between physician and patient remains very strong and probably governs compliance, which is the key to success with ARV treatments just as with other pathologies [29]. Personalisation of a treatment may be called into question by substitution; giving certain patients the impression their treatment is no longer "tailor-made"[30].

Rather than the patients' profiles, it is the theoretical information they have or their personal experience of what must be accepted and the results to expect which appear to play the major role [25]. Substitution of ARV generics in place of brand name medications may well be promoted by HIV specialist physicians who have no fears about their chemical entity, but it appears that the patients' wishes remain a decisive factor on their acceptability. We feel that efficient information about ARV generics not only for physicians but also for patients and patients' associations is an essential step towards improving the acceptability of generic ARVs, which are prescribed and effective in the majority of HIV patients in the world.

## References

[1] De Plazaola JP, Lahi G (2014) Tableau de l’économie Française. Available: http://www.insee.fr/fr/themes/document.asp?ref_id=T14F094. Accessed April 2014.

[2] Projet de loi de financement de la Sécurité sociale PLFSS (2014) Available: www.securite-sociale.fr/IMG/pdf/plfss14-annexe7.pdf. Accessed 1 January 2014.

[3] Guideline on the investigation of bioequivalence (2010) Available: www.ema.europa.eu/docs/en_GB/document_library/Scientific_guideline/2010/01/WC500070039.pdf CPMP/EWP/QWP/1401/98 Rev. 1/Corr. Accessed 20 January 2010.

[4] New EMA guideline on the investigation of bioequivalence (2010) Available: http://www.gmp-compliance.org/enews_02285_New-EMA-Guideline-on-the-Investigation-of-Bioequivalence.html. Accessed 8 December 2010.

[5] World Health Organisation (2010) Health systems financing: The path to universal coverage. The World health report 2010 Geneva. 10.2471/BLT.10.078741 PMC2878164 20539847

[6] ANSM (2012) Les médicaments génériques: des médicaments à part entière. Available: http://www.medicamentsgeneriques.info/wp-content/uploads/2009/12/Ansm_Rapport-Generiques_Decembre2012.pdf. Accessed December 2012.

[7] Shrank WH, Hoang T, Ettner SL, Glassman PA, Nair K, et al (2006) The implications of choice: Prescribing generic or preferred pharmaceuticals improves medication adherence for chronic conditions. Arch Intern Med 166: 332–7. 16476874 10.1001/archinte.166.3.332

[8] Dray-Spira R, Wilson d’Almeida K, Aubrière C, Marcellin F, Spire B, et al (2013) État de santé de la population vivant avec le VIH en France métropolitaine en 2011 et caractéristiques des personnes récemment diagnostiquées. Premiers résultats de l’enquête ANRS-Vespa2. Bull Epidémiol Hebd 26–27: 285–92. 10.3945/jn.111.146258 24737153

[9] Hassali MA, Shafie AA, Jamshed S, Ibrahim MI, Awaisu A (2009) Consumers’ views on generic medicines: A review of the literature. Int J Pharm Pract 17: 79–88. 20214255

[10] Les médecins généralistes Gemme (2010) Available: www.medicamentsgeneriques.info/ http://www.medicamentsgeneriques.info/wp-content/uploads/2010/03/ENQUETE_MEDECINS_GEMME.pdf. Accessed 17 March 2010.

[11] Lagarce L, Lusson-Brisset C, Bruhat C, Diquet B, Lainé-Cessac P (2005) Médicaments génériques, le point de vue des médecins: enquête d’opinion réalisée auprès des médecins libéraux du Maine-et-Loire. Thérapie 60(Suppl.): 67–74. 10.3928/1081597X-20141218-08 15929476

[12] Granlund D (2009) Are private physicians more likely to veto generic substitution of prescribed pharmaceuticals? Soc Sci Med 69: 1643–50. 10.1016/j.socscimed.2009.09.016 19815322

[13] Académie nationale de pharmacie Rapport (2012) Available: http://www.acadpharm.org/dos_public/RAPPORT_GEnEriques_VF_2012.12.21.pdf. Accessed 25 July 2012.

[14] Joel Menkes C (2012) Place des génériques dans la prescription. Bull. Acad. Natle Méd 196, no 2: 521–528.

[15] Collet JP, Silvain J, Cayla G, Bellemain-Appaix A, Montalescot G (2010) Antiagrégants plaquettaires oraux en pratique quotidienne. La lettre du Cardiologue 341: 16–22.

[16] Ringuier R, Rouquette A, Dagorne C, Garnier F, Fanello S (2008) Connaissance et perceptions des médicaments génériques après 50 ans. Thérapie 63 (1): 11–17. 10.3928/1081597X-20141218-08 18387271

[17] Allenet B, Guignon AM, Maire P, Calop J (2005) Intégration des représentations de la personne âgée face à ses médicaments pour améliorer son observance. J Pharm Clin 24(Suppl.): 175–9.

[18] Sarradon-Eck A, Blanc MA, Faure M (2007) Des usagers sceptiques face aux médicaments génériques Une approche anthropologique. Rev Epidemiol Sant Pub 55: 179–85. 10.1016/j.respe.2007.01.026 17459630

[19] Zucman D, Camara S, Gravissea J, Dimia S, Vassea M, et al (2013) Generic antiretroviral drugs in developing countries: friends or foes. AIDS 28: 607–9. 10.1097/QAD.0000000000000170 24378755

[20] Allenet B, Golay A (2013) Quelles sont les attitudes des patients vis-à-vis des médicaments génériques? Illustration par la metformine. Rev Med Suisse 9: 1005–1009. 23750394

[21] Shrank WS, Stedman M, Ettner SL, DeLapp D, Dirstine J, et al (2007) Patient, physician, pharmacy, and pharmacy benefit design factors related to generic medication use. J Gen Intern Med 22:1298–304. 17647066 10.1007/s11606-007-0284-3 PMC2219782

[22] Chong C, March G, Clark A, Gilbert A, Hassali MA, et al (2011) A nationwide study on generic medicines substitution practices of Australian community pharmacists and patient acceptance. Health Policy 99: 139–48. 10.1016/j.healthpol.2010.08.002 20732723

[23] Vallès JA, Barreiro M, Cereza G, Ferro JJ, Martínez MJ, et al (2003) A prospective multicenter study of the effect of patient education on acceptability of generic prescribing in general practice. Health Policy 65: 269–75. 12941494 10.1016/s0168-8510(03)00018-6

[24] Kjoenniksen I, Lindbaek M, Granas AG (2006) Patients’ attitudes towards and experiences of generic drug substitution in Norway. Pharm World Sci 28: 284–9. 17111247 10.1007/s11096-006-9043-5

[25] Figueiras M, Marcelino D, Cortes M (2008) People’s views on the level of agreement of generic medicines for different illnesses. Pharm World Sci 30: 590–4. 10.1007/s11096-008-9247-y 18704749

[26] Barrett LL (2005) Physicians’ attitudes and practices regarding generic drugs. Availabe: http://assets.aarp.org/rgcenter/health/phys_generic.pdf. Accessed 14 February 2008.

[27] Allenet B, Sailly JC (1999) La mesure du bénéfice en santé par la méthode du consentement à payer. J Econ Med 17(Suppl.): 301–26.

[28] Decollogny A, Eggli Y, Halfon P, Lufkin TM (2011) Determinants of generic drug substitution in Switzerland. BMC Health Services Research 11:17 www.biomedcentral.com/1472-6963/11/17. 10.1186/1472-6963-11-17 21269426 PMC3038892

[29] Hakonsen H, Eilertsen M, Borge H, Toverud E (2009) Generic substitution: Additional challenge for adherence in hypertensive patients? Curr ed Res Opin 25: 2515–21. 10.1185/03007990903192223 19708764

[30] Baudrant-Boga M, Allenet B (2011) Inertie thérapeutique: et le patient dans tout ça? Médecine des maladies métaboliques 5: S73–80 10.1038/nutd.2014.45 25599560 PMC4314578

